# MMP-1 serum levels predict coronary atherosclerosis in humans

**DOI:** 10.1186/1475-2840-8-50

**Published:** 2009-09-14

**Authors:** Michael Lehrke, Martin Greif, Uli C Broedl, Corinna Lebherz, Rüdiger P Laubender, Alexander Becker, Franz von Ziegler, Janine Tittus, Maximilian Reiser, Christoph Becker, Burkhard Göke, Gerhard Steinbeck, Alexander W Leber, Klaus G Parhofer

**Affiliations:** 1 Department of Internal Medicine II, University of Munich, Munich, Germany; 2 Department of Internal Medicine I, University of Munich, Munich, Germany; 3 Institute for Medical Informatics, Biometry and Epidemiology (IBE), University of Munich, Munich, Germany; 4 Department of Radiology, University of Munich, Munich, Germany

## Abstract

**Background:**

Myocardial infarction results as a consequence of atherosclerotic plaque rupture, with plaque stability largely depending on the lesion forming extracellular matrix components. Lipid enriched non-calcified lesions are considered more instable and rupture prone than calcified lesions. Matrix metalloproteinases (MMPs) are extracellular matrix degrading enzymes with plaque destabilisating characteristics which have been implicated in atherogenesis. We therefore hypothesised MMP-1 and MMP-9 serum levels to be associated with non-calcified lesions as determined by CT-angiography in patients with coronary artery disease.

**Methods:**

260 patients with typical or atypical chest pain underwent dual-source multi-slice CT-angiography (0.6-mm collimation, 330-ms gantry rotation time) to exclude coronary artery stenosis. Atherosclerotic plaques were classified as calcified, mixed or non-calcified.

**Results:**

In multivariable regession analysis, MMP-1 serum levels were associated with total plaque burden (OR: 1.37 (CI: 1.02-1.85); p < 0.05) in a model adjusted for age, sex, BMI, classical cardiovascular risk factors, hsCRP, adiponectin, pericardial fat volume and medication. Specification of plaque morphology revealed significant association of MMP-1 serum levels with non-calcified plaques (OR: 1.16 (CI: 1.0-1.34); p = 0.05) and calcified plaques (OR: 1.22 (CI: 1,03-1.45); p < 0.05) while association with mixed plaques was lost in the fully adjusted model. No associations were found between MMP9 serum levels and total plaque burden or plaque morphology.

**Conclusion:**

MMP-1 serum levels are associated with total plaque burden but do not allow a specification of plaque morphology.

## Introduction

Myocardial infarction is an acute pathological event which is caused by the rupture of a chronically formed atherosclerotic lesion. The process of atherosclerosis is initiated by the entrapment of modified LDL particles in the subendothelial space of the vessel wall, which causes the infiltration of immune cells, especially macrophages and subsequent foam cell formation. Proliferation of vascular smooth muscle cells (VSMC) and the deposition of extracellular matrix components result in the progression to more advanced lesions, stigmatized by a fibrous cap overlaying a lipid enriched atherosclerotic core [1]. Plaque rupture mostly occurs in the plaques' shoulder region, which is exposed to high tangential shear stress. The biochemical strength of the fibrous cap thereby depends on its extracellular matrix composition with fibrillar collagen, especially type I and III, providing structural stability by cross linkage [2].

Intravascular ultrasound (IVUS) and CT angiography do now allow the in-vivo detection of outward directed non-stenosing lesions in addition to the characterisation of plaque morphology. Lipid enriched, non-calcified lesions are primarily detected in the coronary system of patients presenting with unstable angina, suggesting a high rupture susceptibility of these plaques, while fibrous and calcified lesions dominate in patients presenting with stable angina [3-6]. This concept is supported by autopsy studies demonstrating impaired structural stability of soft, lipid enriched plaques in comparison to calcified lesions [7]. Consequently, non-calcified lesions were prospectively associated with higher coronary event rates [8,9].

Matrix Metalloproteinases (MMPs) are a family of extracellular matrix degrading enzymes which have been implicated in plaque destabilisation [10]. MMPs are expressed by macrophages, VSMC and endothelial cells in response to inflammatory stimuli. MMP-1 and MMP-9 are primarily detected in the vulnerable shoulder region and areas of foam cell formation in the atherosclerotic plaques [10,11] where they colocalize with degraded collagen fragments [11]. While MMP-1 cleaves intact fibrillar interstitial collagen, especially type I and III, MMP-9 has prominent activity against basement membrane components including type IV collagen, laminin and elastin [12]. Consistently, polymorphisms of the MMP-1 and -9 genes were linked to complicated coronary lesions and atherosclerosis in epidemiological studies [13,14].

We therefore hypothesized MMP-1 and MMP-9 serum levels to predict the presence of non-calcified, lipid enriched plaques as determined by CT angiography in a cohort of 260 patients with typical or atypical chest pain.

## Methods

### Ascertainment of Subjects

As described previously [15], 260 patients with typical or atypical chest pain underwent dual-source CT-coronary angiography for exclusion of coronary artery stenosis during a period of 20 months from March 2006 till October 2007. Study subjects were asked to complete a brief questionnaire and had blood drawn after providing written informed consent. The study protocol was approved by the Ethics Committee of the Ludwig-Maximilians-University Munich, Germany.

### Dual-source CT image analysis

CT-coronary angiography was performed as described previously [15], using a Siemens Definition scanner (Siemens Medical Solutions, Forchheim, Germany). Briefly, DSCT datasets were evaluated by two independent investigators using a dedicated cardiac workstation (Siemens, Leonardo Circulation). Atherosclerotic plaques were classified as calcified, mixed or non-calcified lesions. Calcified plaques were defined as lesions with a HU value above 130 while non-calcified plaques were defined as structures clearly assignable to the vessel wall (in at least two views) with densities less than the lumen contrast. Mixed plaques where defined by < 50% plaque calcified area. The coronary tree was segmented according to the suggestions of the AHA into a 15 segment model. Each segment was further divided into a proximal and a distal segment. Each segment was then classified as containing either calcified, non-calcified, mixed or no plaque. Based on the number of diseased segments a plaque score was calculated.

Pericardial fat assessment was performed as described previously using the Volume analysis software tool of the Siemens Leonardo Circulation workstation [16].

### Laboratory procedures

Blood samples were stored at -70°C until analysis. Serum levels of MMP-1 and MMP-9 were determined using a commercial enzyme-linked immunosorbent assay (R&D, Wiesbaden, Germany) following the manufacturer's instruction. Intra- and interassay coefficients of variance derived from a pooled human plasma sample measured in triplicate on each plate were 4.1% and 6.7% for MMP-1 and 3.5% and 5.9% for MMP-9. Adiponectin serum levels were assessed by enzyme-linked immunosorbent assay (R&D, Wiesbaden, Germany) as described earlier [15]. Plasma total cholesterol, LDL-cholesterol (LDL-C), HDL-cholesterol (HDL-C) and triglycerides were measured by routine enzymatic methods. Determination of high sensitivity C-reactive protein (hsCRP) levels was performed at the Department of Clinical Chemistry (Campus Grosshadern, University of Munich, Germany).

### Statistical Analysis

Statistical analyses were performed using SPSS 16 and R (version 2.8.1) software. (R Development Core Team (2008). R: A language and environment for statistical computing. R Foundation for Statistical Computing, Vienna, Austria. ISBN 3-900051-07-0, URL .)

Data are reported as proportions or median (interquartile range). Spearman correlation was perfomed as appropriate to evaluate unadjusted associations between MMP-1 or MMP-9 and other variables. A generalized linear regression model (logistic regression) based on complete cases for all covariables was used to assess the association between MMP-1 and MMP-9 serum levels with total plaque burden or the presence of calcified-, mixed- or non-calcified plaques adjusted for age, sex, body mass index (BMI), diabetes, hypertension, family history of coronary artery disease, smoking, LDL-C, HDL-C, triglycerides, hsCRP levels, adiponectin levels, medical treatment (statin, ACE inhibitors, angiotensin-receptor blockers, diuretics, ASS, Clopidogrel, Phenprocoumon) and pericardial adipose tissue volume.

### Results

**Figure 1:**
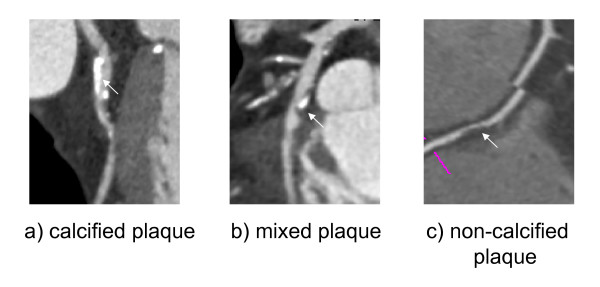
**Representative images of calcified (a), mixed (b) and non-calcified plaques (c) as detected by CT-angiography**.

The baseline characteristic of the study sample is presented in Table 1. Representative images of different plaque morphologies as detected by CT-angiography are shown in Figure 1.

**Table 1:** Characteristics of the study population:

**Characteristics:**	**n = 260**
**Age (yrs)**	63 (54 - 69)
**Men (%)**	66.8
**Classical CV-Risk Factors (%)**	
Diabetes*	6.2
Hypertension*	47.3
Family history of CAD**	26.9
Smoking**	15
**Body Mass Index (kg/m2)**	26.2 (24.2 - 29.3)
**Laboratory profile**	
Total cholesterol (mg/dl)	209 (181.2 - 239)
LDL cholesterol (mg/dl)	125 (96.7 - 151)
HDL cholesterol (mg/dl)	52 (44 - 60)
Triglycerides (mg/dl)	141 (103 - 197.5)
C-reactive protein (mg/dl)	0.23 (0 - 0.53)
Adiponectin (ug/ml)	5.05 (3.25 - 7.67)
MMP-1 (ng/ml)	3.91 (2.24 - 5.67)
MMP-9 (ng/ml)	211.3 (147.1 - 283)
Medical treatment (%)***	
Statin	37.7
Anticoagulation/Antiplatelet	63.5
Betablocker	64.9
ACE-I or ARB	51.8
Diuretics	30.6
**CT data**	
Pericardial adipose tissue in volume (ml)§	184.1 (128.6 - 255.8)
Number of coronary artery plaques (total)§§	3 (1 - 6)
Number of calcified plaques (154 patients)	1 (0 - 3)
Number of mixed plaques (102 patients)	0 (0 - 1)
Number of non-calcified plaques (126 patients)	0 (0 - 2)

Anticoagulation: Aspirin, Clopidogrel, Phenprocoumon

Values are presented as proportions or median (interquartile range). Minimum/maximum values are presented for plaque data. No plaques were found in 51 patients.

* history of hypertension and diabetes is known in 236 patients

** history of smoking and family history of CAD is known in 235 patients

*** medical treatment is known in 222 patients

§adequate image quality for evaluation of PAT was obtained in 248 patients

§§ adequate image quality for evaluation of coronary plaques was obtained in 242 patients

### Association of MMP-1 and MMP-9 serum levels with cardiovascular risk factors and coronary atherosclerosis

MMP-1 and MMP-9 were found to correlate positively to the inflammatory marker hsCRP (MMP-1, r = 0.194, p < 0.001; MMP-9, r = 0.173, p = 0.005) and to each other (r = 0.269; p < 0.001) (Table 2). In addition, positive correlation of both MMPs were found with serum triglycerides (MMP-1, r = 0.195, p = 0.002; MMP-9, r = 0.135, p = 0.03), while only MMP-9 correlated to BMI (r = 0.149; p = 0.02) and pericardial adipose tissue volume (r = 0.253; p < 0.001) (Table 2). '

**Table 2:** Correlation of Serum MMP-1 and MMP-9 levels with cardio vascular risk factors and plaque morphology

		**MMP-1**		**MMP-9**	
		**Median (IQR)**	**P**	**Median (IQR)**	**P**
		**Rho**		**Rho**	

**Age**		-0.003	0.97	0.004	0.50

**Sex**					
**- female**		4.46 (2.24 - 5.69)	0.6	207 (137-169)	0.16
**- male**		3.38 (2.12 - 5.52)		220 (160-288)	

**BMI**		0.025	0.69	0.149	**0.018**

**Diabetes mellitus:**	**Yes**	4.34 (2.99 - 5.82)	0.60	243.2 (189.9-354.1)	0.89
	**No**	3.78 (2.09 - 5.67)		209.6 (144.8-277.2)	
**Hypertension:**	**Yes**	3.74 (2.10 - 5.82)	0.89	212.7 (150.1 - 288.1)	0.67
	**No**	3.96 (2.23 - 5.67)		195.6 (135.5 - 266.9)	
**Family history of CAD:**	**Yes**	3.73 (1.80 - 5.64)	0.45	195.6 (140.4 - 268.4)	0.15
	**No**	3.93 (2.24 - 5.69)		212.4 (148.1 - 287.5)	
**Smoking:**	**Yes**	4.08 (1.90 - 5.70)	0.86	185.6 (133.6 - 283.2)	0.97
	**No**	3.73 (2.24 - 5.67)		212.3 (146.0 - 275.8)	
**Statin treatment:**	**Yes**	4.11 (2.44 - 5.90)	0.25	205.2 (138.8 - 284.1)	0.44
	**No**	3.52 (2.09 - 5.65)		212.3 (153.7 - 280.6)	
**Anticoagulation:**	**Yes**	3.71 (2.18 - 5.65)	0.32	180.9 (137.0 - 257.5)	0.06
	**No**	3.67 (2.02 - 5.61)		235.0 (157.6 - 313.5)	
**Betablocker:**	**Yes**	3.91 (2.44 - 5.23)	0.80	206.5 (139.5 - 273.5)	**0.049**
	**No**	3.48 (2.07 - 6.07)		217.8 (158.5 - 339.3)	
**ACE-I or ARB:**	**Yes**	4.11 (2.44 - 5.65)	0.51	207.6 (144.7 - 283.2)	0.66
	**No**	3.5 (2.09 - 5.82)		210.3 (153.7 - 282.3)	
**Diuretics:**	**Yes**	4.06 (2.90 - 5.59)	0.32	211.8 (141.3 - 300.0)	0.76
	**No**	3.6 (2.07 - 5.79)		209.6 (151.1 - 276.9)	

**Pericardial adipose tissue**		0.068	0.28	0.253	**<0.0001**
**Adiponectin**		-0.111	0.08	-0.084	0.18
**High-sensitivity CRP**		0.194	**0.001**	0.173	**0.005**
**Triglycerides**		0.195	**0.002**	0.135	**0.028**
**Total-cholesterol**		0.041	0.52	-0.037	0.53
**LDL-cholesterol**		-0.002	0.97	-0.049	0.43
**HDL-cholesterol**		-0.048	0.45	-0.018	0.77
**MMP-1**				0.269	**<0.0001**

**Total-Plaque burden**		0.16	**0.013**	0.087	0.17
**Non-Calcified Plaques**		0.157	**0.014**	0.002	0.98
**Mixed Plaques**		0.119	0.07	0.12	0.06
**Calcified Plaques**		0.118	0.07	0.058	0.37

Median (IQR) or Spearman correlation coefficient are presented. (CAD: Coronary artery disease)

**Figure 2:**
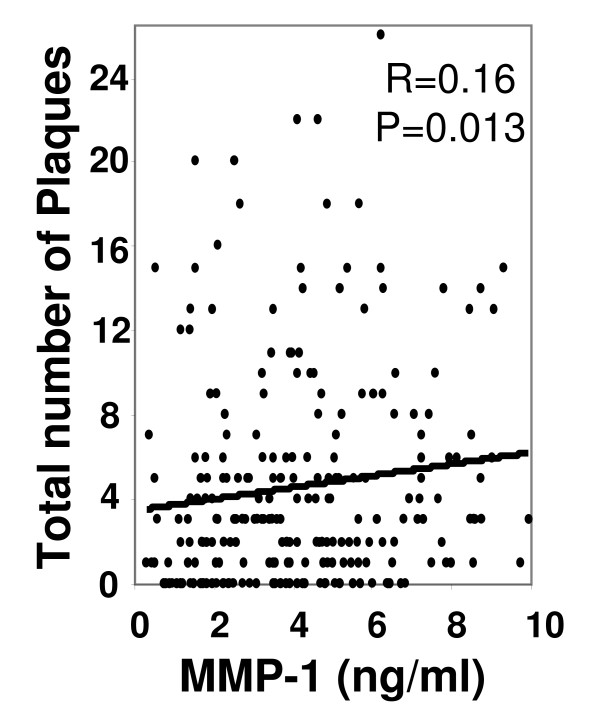
**Correlation between MMP-1 serum levels and total plaque burden**.

MMP-1 but not MMP-9 serum levels correlated with total plaque burden (r = 0.16; p = 0.013) in addition to non-calcified lesions (r = 0.157; p = 0.014) (Figure 2, Table 2).

No associations of either parameter were found with age, sex, total cholesterol, LDL-cholesterol, HDL-cholesterol, adiponectin, classical cardiovascular risk factors or medication, although patients receiving betablockers had lower MMP-9 levels (p = 0.049)(Table 2).

### MMP-1 serum level predict total plaque burden

In multivariable logistic regression analysis MMP-1 serum levels were associated with total plaque burden in a simple model adjusting for age, sex, BMI (OR 1.28 (CI: 1.02 to 1.59; p < 0.05) as well as in more complex models adjusting for all covariables (age, sex, BMI, classical cardiovascular risk factors, hsCRP, adiponectin, pericardial fat volume, medication) (OR 1.37 (CI: 1.02 to 1.85; p < 0.05) (Table 3). No association of MMP-9 was found with total plaque burden (Table 3).

**Table 3:** Multivariate Association of MMP-1 and MMP-9 serum levels and total plaque burden as assessed by CT-angiography

**Total Plaque**	**MMP-1**		**MMP-9**	
**Adjusted for**	**OR (CI)**	**P value**	**OR (CI)**	**P value**

**Age, Gender, BMI**	1.28 (1.02 to 1.59)	**0.031**	0.97 (0.90 to 1.05)	0.49
Age, Gender, BMI, RF*	1.30 (1.01 to 1.66)	**0.040**	0.96 (0.90 to 1.03)	0.29
Age, Gender, BMI, RF*, CRP	1.29 (1.00 to 1.67)	**0.044**	0.96 (0.90 to 1.03)	0.28
Age, Gender, BMI, RF*, CRP, AD, PCF	1.32 (1.01 to 1.72)	**0.040**	0.97 (0.90 to 1.03)	0.32
Age, Gender, BMI, RF*, CRP, AD, PCF, Med	1.37 (1.02 to 1.85)	**0.037**	0.98 (0.90 to 1.06)	0.63

Odds ratio and 95% CI increase in plaque quantity for 1 ng/ml (MMP-1) and 20 ng/ml (MMP-9) raise in serum levels are presented.

*Risk factors (RF*) included LDL-cholesterol, HDL-cholesterol, triglycerides, cigarette smoking, hypertension, diabetes, family history for CVD.

Abbreviations: CRP (hsCRP), AD (adiponectin), PCF (pericardial fat volume), Med (medication)

### MMP-1 serum levels predict calcified and non-calcified lesions

To further evaluate the relevance of MMP-1 and MMP-9 serum levels to predict the presence of calcified, mixed or non-calcified plaques, a multivariable logistic regression analysis was performed for each parameter. Positive association of MMP-1 serum levels were found with calcified (OR 1.22 (CI: 1.04 to 1.43; p < 0.05), mixed (OR 1.19 (CI: 1.03 to 1.38; p < 0.049), and non-calcified lesions (OR 1.19 (CI: 1.04 to 1.37; p < 0.05) after adjusting for age, sex, BMI and classical cardiovascular risk factors (Table 4). This association remained significant for calcified (OR 1.22 (CI: 1.03 to 1.45; p < 0.05) and non-calcified (OR 1.16 (CI: 1.0 to 1.34; p = 0.05) lesions after further adjustment for hsCRP, adiponectin, pericardial fat volume and medication but was lost for mixed lesions (Table 4). No association was found between MMP-9 serum levels and non-calcified, mixed or calcified lesions.

**Table 4:** Multivariate Association of MMP-1 and MMP-9 serum levels and quantity of non-calcified, mixed and calcified plaques as assessed by CT-angiography



**Non-calcified Plaque**	**MMP-1**		**MMP-9**	

**Adjusted for**	**OR (CI)**	**P value**	**OR (CI)**	**P value**

**Age, Gender, BMI**	1.19 (1.04 to 1.37)	**0.012**	0.99 (0.95 to 1.04)	0.74
Age, Gender, BMI, RF*	1.19 (1.03 to 1.37)	**0.017**	0.99 (0.95 to 1.04)	0.76
Age, Gender, BMI, RF*, CRP	1.20 (1.04 to 1.38)	**0.014**	1 (0.95 to 1.05)	0.90
Age, Gender, BMI, RF*, CRP, AD, PCF	1.18 (1.03 to 1.37)	**0.021**	0.99 (0.94 to 1.04)	0.80
Age, Gender, BMI, RF*, CRP, AD, PCF, Med	1.16 (1.00 to 1.34)	**0.050**	1 (0.95 to 1.05)	0.90

**Mixed Plaque**	**MMP-1**		**MMP-9**	

**Adjusted for**	**OR (CI)**	**P value**	**OR (CI)**	**P value**

**Age, Gender, BMI**	1.19 (1.03 to 1.38)	**0.019**	0.99 (0.94 to 1.04)	0.71
Age, Gender, BMI, RF*	1.19 (1.02 to 1.39)	**0.014**	0.99 (0.94 to 1.04)	0.77
Age, Gender, BMI, RF*, CRP	1.19 (1.02 to 1.39)	**0.024**	0.99 (0.94 to 1.05)	0.79
Age, Gender, BMI, RF*, CRP, AD, PCF	1.17 (1.00 to 1.37)	**0.047**	0.99 (0.94 to 1.04)	0.67
Age, Gender, BMI, RF*, CRP, AD, PCF, Med	1.14 (0.96 to 1.35)	0.12	0.99 (0.94 to 1.05)	0.83

**Calcified Plaque**	**MMP-1**		**MMP-9**	

**Adjusted for**	**OR (CI)**	**P value**	**OR (CI)**	**P value**

**Age, Gender, BMI**	1.22 (1.04 to 1.43)	**0.013**	0.99 (0.94 to 1.05)	0.78
Age, Gender, BMI, RF*	1.22 (1.04 to 1.43)	**0.016**	0.98 (0.93 to 1.04)	0.69
Age, Gender, BMI, RF*, CRP	1.21 (1.03 to 1.42)	**0.020**	0.98 (0.93 to 1.04)	0.49
Age, Gender, BMI, RF*, CRP, AD, PCF	1.21 (1.03 to 1.43)	**0.019**	0.98 (0.94 to 1.05)	0.57
Age, Gender, BMI, RF*, CRP, AD, PCF, Med	1.22 (1.03 to 1.45)	**0.023**	0.98 (0.9 w to 1.05)	0.64

Odds ratio and 95% CI increase in plaque quantity for 1 ng/ml (MMP-1) and 20 ng/ml (MMP-9) raise in serum levels are presented.

*Risk factors (RF*) included LDL-cholesterol, HDL-cholesterol, triglycerides, cigarette smoking, hypertension, diabetes, family history for CVD.

Abbreviations: CRP (hsCRP), AD (adiponectin), PCF (pericardial fat volume), Med (medication)

## Discussion

In this study we report a positive association of MMP-1 serum levels with total plaque burden in a population of 260 patients who underwent CT-angiography for exclusion of coronary artery disease.

We thereby found MMP-1 serum levels to associate with calcified lesions and non-calcified lesions after adjustment for age, sex, BMI, classical cardiovascular risk factors, hsCRP, adiponectin, pericardial fat volume and medication. MMP-1 serum levels were further associated with mixed lesions in reduced statistical models, which was however lost after adjustment for all covariables.

Clinical risk management of cardiovascular disease would greatly benefit from event stratifying biomarkers. The rupture prone atherosclerotic plaque is histologically defined by a thin fibrous cap overlaying a lipid enriched atheromatous core [1]. Non-calcified, lipid enriched plaques have primarily been found in patients presenting with acute coronary syndrome and unstable angina, while mixed or calcified lesions dominated in patients with stable angina [3-6]. Consequently, non-calcified plaques were prospectively associated with increased coronary event rates [8,9], and short term tendency for plaque volume progression [17]. Non-calcified plaques have therefore been considered to be more prone to rupture than calcified lesions. Consistently, we did find MMP-1 serum levels to be positively associated with non calcified lesions, however, similar associations were found with calcified- and to a lesser extent with mixed lesions, resulting in a positive association of MMP-1 serum levels with total plaque burden without discriminative power for a specific plaque morphology.

Evidence for plaque destabilising characteristics of MMP-1 has been created by its accumulation in the vulnerable shoulder region of thin capped, lipid enriched plaques and its co-localization with degraded collagen fragments [10,11]. In addition, increased serum levels of MMP-1 have been found in patients with coronary artery disease in some [18,19], but not all studies [20], with highest levels being present in patients with unstable angina [21]. The positive association of MMP-1 serum levels with total plaque burden as reported in our study therefore supports the concept of MMP-1 being significantly released from atherosclerotic plaques. The missing discriminative power of MMP-1 serum levels for a specific plaque morphology however suggests MMP-1 to be similarly present within all plaques, independent of their morphology, questioning its relevance as a major plaque destabilizing factor. Alternatively, non-calcified lesions as determined by CT-angiography might not sufficiently identify the rupture prone vulnerable lesion. CT-angiography has been reported to provide a diagnostic sensitivity of 82 to 84% and specificity of 88 to 91% in comparison to IVUS as the gold standard [22,23]. Highest diagnostic accuracy was found for calcified lesions (sensitivity 94% to 95%; specificity 92% to 94%), while non-calcified lesions were detected with a diagnostic sensitivity of 78% by our group in a previous study and 53% by Achenbach et al. [22,23]. In addition, CT-angiography has limited capacities to further subdivide non-calcified lesions in fibrous and lipid enriched plaques. CT-angiography therefore provides an advanced non-invasive diagnostic tool for the detection of coronary plaques, which however still holds modest limitations in the detection of non-calcified lesions. We can not rule out that this might have affected the presented results.

MMP-1 and MMP-9 are primarily expressed by macrophages and smooth muscle cells in response to a variety of proatherogenic stimuli including sheer stress, oxidized LDL and inflammatory cytokines [10]. Consistently, circulating MMP-1 levels have been associated with atherosclerotic plaque inflammation as detected by 18F-FDG-PET/CT [18]. In agreement with their proinflammatory regulation we found both MMPs to correlate positively with the inflammatory marker hsCRP. Our study therefore supports the concept of an inflammatory microenvironement characterising the atherosclerotic plaque which potentially could reflect plaque macrophage content. In contrast to other studies we could not ascribe highest inflammatory activity to non-calcified lesions [24,25]. Future studies are needed to characterise plaque morphology dependent inflammation in-vivo, preferentially by combining CT-Angiography with 18F-FDG-PET.

Surprisingly, we did not find associations between MMP-9 serum levels and total plaque burden or plaque morphology. This agrees with some reports [26,27] but contrasts to others, which found incremental MMP-9 serum levels in patients with coronary artery disease [28,29]. The reason for this discrepancy is not clear but might be related to different patient populations and a differential assessment of atherosclerosis. CT angiography as used in our study allows the assessment of non-stenosing, outward directed lesions of the vessel wall and is not limited to the detection of stenosing lesions as assessed by coronary angiography [30].

Interestingly and consistent with others we found positive association between MMP-9 serum levels and BMI [31,32], in addition to pericardial fat volume and serum triglyceride concentrations, suggesting adipose tissue to be a significant determinant of circulating MMP-9 levels.

Our study has several limitations. Detection of coronary plaques quantity and morphology by CT-angiography does not provide the diagnostic accuracy of IVUS as the gold standard. The presented results are associative and can not prove causal relationship between MMP-1 serum levels and atherosclerotic plaque quantity or morphology. In addition, the cross sectional study design did not allow an evaluation of clinical relevance with respect to future cardiac event rates. Future studies are needed to confirm our findings in larger patient cohorts possibly by applying a prospective study design.

In conclusion we found MMP-1 serum levels to be an independent predictor for coronary atherosclerotic lesions, while not allowing a stratification of plaque morphology.

## Competing interests

The authors declare that they have no competing interests.

## Authors' contributions

ML: study design, data analysis, manuscript preparation, MG: study design, data analysis, manuscript editing, UCB: study design, manuscript editing, CL: data acquisition, manuscript editing, RPL: statistical analysis, AB: study design, manuscript editing, FvZ: data analysis, manuscript editing, MR: final approval of the manuscript, CB: data acquisition, BG: final approval of the manuscript, GS: final approval of the manuscript, AWL: study design, manuscript editing, KGP: study design, manuscript editing. All authors read and approved the final manuscript. 
